# Association of Brain CD163 Expression and Brain Injury/Hydrocephalus Development in a Rat Model of Subarachnoid Hemorrhage

**DOI:** 10.3389/fnins.2018.00313

**Published:** 2018-05-16

**Authors:** Chaohui Jing, Haining Zhang, Hajime Shishido, Richard F. Keep, Ya Hua

**Affiliations:** ^1^Department of Neurosurgery, University of Michigan, Ann Arbor, MI, United States; ^2^Department of Neurosurgery, Xinhua Hospital, School of Medicine, Shanghai Jiaotong University, Shanghai, China

**Keywords:** CD163, magnetic resonance imaging, T2 lesion, hydrocephalus, subarachnoid hemorrhage

## Abstract

Hemoglobin contributes to brain cell damage and death following subarachnoid hemorrhage (SAH). While CD163, a hemoglobin scavenger receptor, can mediate the clearance of extracellular hemoglobin it has not been well-studied in SAH. In the current study, a filament perforation SAH model was performed in male rats. T2-weighted and T2^*^-weighted scans were carried out using a 7.0-Tesla MR scanner 24 h after perforation. T2 lesions and hydrocephalus were determined on T2-weighted images. A grading system based on MRI was used to assess SAH severity. The effects of SAH on CD163 were determined by immunohistochemistry staining and Western blots. SAH led to a marked increase in CD163 levels in cortex, white matter and periventricular regions from days 1 to 7. CD163 stained cells were co-localized with neurons, microglia/macrophages, oligodendrocytes and cleaved caspase-3-positive cells, but not astrocytes. Furthermore, CD163 protein levels were increased in rats with higher SAH grades, the presence of T2 lesions on MRI, or hydrocephalus. In conclusion, CD163 expression is markedly upregulated after SAH. It is associated with more severe hemorrhage, as well as MRI T2 lesion and hydrocephalus development.

## Introduction

Spontaneous subarachnoid hemorrhage (SAH) is usually caused by a ruptured intracranial aneurysm. It is a form of stroke with a high mortality rate and early brain injury after SAH is an important factor contributing to death and disability. However, the main mechanisms of the early brain injury are still unknown. A combination of elevated intracranial pressure, transient global ischemia injury, blood-brain barrier disruption, and brain edema may contribute to early brain injury after SAH (Sehba and Bederson, 2006; Lee et al., 2010; Jing et al., 2012).

Hemoglobin released from red blood cells induces cell death and brain injury in intracerebral hemorrhage (ICH) and SAH (Xi et al., 2006; Lee et al., 2010). Such damage may be limited by hemoglobin clearance and degradation. Hemoglobin tightly binds to haptoglobin and haptoglobin/hemoglobin complexes can be taken up by microglia/macrophages via the CD163 receptor (Madsen et al., 2001). Heme oxygenase-1 (HO-1) is a key enzyme of heme degradation and the haptoglobin/hemoglobin-CD163-HO-1 system represents a basic line of defense against hemoglobin neurotoxicity by facilitating hemoglobin removal (Thomsen et al., 2013). We and others have demonstrated that CD163 is expressed and contributes to hematoma clearance following ICH (Cao et al., 2016; Liu et al., 2017; Leclerc et al., 2018), but the functions of CD163 have not been investigated after SAH.

The current study examined the impact of SAH on CD163 in an endovascular perforation model in rats due to similarities in the pathophysiological events observed in that model to those occurring in SAH patients suffering from aneurysmal rupture (Kooijman et al., 2014; Sehba, 2014). Nevertheless, the extent and volume of subarachnoid clot in that model is uncontrollable and varies between animals (Lee et al., 2009). Therefore, we used a new classification system based upon magnetic resonance image (MRI) to assess SAH severity (Shishido et al., 2015). Thus, T2^*^-weighted MRI was performed to measure blood accumulation in the brain after SAH. T2-weighted MRI was used for assessing hydrocephalus occurrence and lesion determination. Western blot and immunohistochemistry was used to assess CD163 expression following SAH.

## Experimental procedures

### Animals and SAH

All animal procedures used in this experiment were approved by the University of Michigan Committee on Use and Care of Animals. The animals were housed with *ad libitum* food and water. In total, 76 male Sprague-Dawley rats (250-350 g) purchased from Charles River Laboratories were used in this study. The filament perforation model was performed as previously described (Lee et al., 2010; Okubo et al., 2013). In brief, rats were anesthetized by 5% isoflurane and later isoflurane concentration was titrated at 2.5-3% after intubation. A feedback controlled heating pad was used to maintain rat rectal temperature at 36.5°C during surgical procedures. The left carotid artery and its branches were identified under a surgical microscope and the external carotid artery (ECA) was transected. A 3-0-nylon suture was entered through the stump of ECA into the internal carotid artery until it reached and perforated the bifurcation of intracranial internal carotid artery. The ECA was tightened to prevent blood loss after the nylon suture was withdrawn. Sham-operated rats underwent the same surgical procedure, but the nylon filament was not introduced far enough to perforate the internal carotid artery. The mortality rate was 24% (16/68) after SAH; no sham operated rats died (*n* = 8). Therefore, 40 SAH rats and 8 sham rats were euthanized at day 1, and 12 SAH rats were euthanized at day 7 for either immunohistochemistry or Western blots.

### MRI measurements and grading

Twenty-four hours after SAH induction, MRI was performed in a 7.0 Tesla Varian MR scanner (Varian Inc., Palo Alto, California) including T2 fast spin-echo and T2^*^ gradient-echo sequences. The chosen MRI parameters were: a field of view = 35 × 35 mm, matrix = 256 × 256 mm, and slice thickness = 0.5 mm. T2 lesions in the ipsilateral brain were identified when the pixel value was over 2 standard deviations (SD) from the mean in the contralateral brain. Measurements of ventricular volume were determined as described previously (Okubo et al., 2013). Hydrocephalus was identified when the ventricular volume following SAH was more than 3 SD above the mean in the control group.

### SAH grading in MRI

The rat perforation model produces different degrees of SAH and a bleeding scale, previously described by Shishido et al. (2015), was used to assess SAH severity. In brief, the MRI grades for perforation SAH model were as follows: grade 0: no SAH or intraventricular hemorrhage (IVH), grade 1: minimal or thin SAH without IVH, grade 2: minimal or thin SAH with IVH, grade 3: thick SAH without IVH, and grade 4 thick SAH with IVH. IVH was defined as at least one hypo-intensity clot recognized in any ventricle on T2^*^ imaging. Thick SAH was defined as at least two slices with clots thicker than 0.5 mm on T2^*^ MRI.

### Immunohistochemistry

Immunohistochemistry was performed using the avidin-biotin-peroxidase complex techniques as previously reported (Jing et al., 2012; Liu et al., 2017). Rats were anesthetized with a lethal sodium dose of pentobarbital and underwent transcardiac perfusion with 4% paraformaldehyde. Subsequently, the brains were harvested, post-fixed in 4% paraformaldehyde overnight, and cryo-protected in 30% sucrose at 4°C. OCT compound embedded brains were sliced into 18 μm sections with a cryostat microtome, dried and preserved at −80°C. The primary antibody was mouse-anti-CD163 (AbD, MCA2311, 1:400). The secondary antibody was biotinylated horse-anti-mouse IgG (1:500, BA2001, Vector). Negative controls were executed by incubating with normal horse serum.

### Immunofluorescence labeling

For triple labeling, the primary antibodies were mouse-anti-CD163 (1:400, MCA2311, AbD), rabbit-anti-CD163 (1:300, ab87099, Abcam), mouse-anti-NeuN (Abcam, ab104225, 1:500), goat-anti-GFAP (1:2,000, ab53554, Abcam); goat-anti-IBA-1 (1:2,000, ab107159, Abcam); mouse-anti-CC1 (1:400, 2869730, Novus Biologicals) and rabbit-anti-cleaved caspase-3 (1:500, 9,661, CST). The secondary antibodies(Invitrogen) used in this study included Alexa Fluor 488-labeled (green channel) donkey-anti-mouse IgG, Alexa Fluor 488-labeled (green channel) donkey-anti-rabbit IgG, Alexa Fluor 594-labeled (red channel) donkey-anti-rabbit IgG, Alexa Fluor 594-labeled (red channel) donkey-anti-mouse IgG, and Alexa Fluor 594-labeled (red channel) donkey-anti-goat IgG. All the secondary antibodies used were diluted to 1:500. Cell nuclei were stained using Fluoroshield containing DAPI (blue channel, F6057, Sigma).

### Western blots

Western blots were undertaken as previously described for rats after SAH (Cao et al., 2016). Rats were anesthetized and underwent transcardiac perfusion with phosphate-buffered saline (PBS, 0.1 mmol/L, pH 7.4) for 5 min. Brain hemispheres were coronally cut into a section of 3-mm thickness through the center of optic chiasm, and the ipsilateral and contralateral cortex separated. Tissue was homogenized in lysis buffer and the protein concentration measured. Fifty μg protein was separated and transferred to hybond-C pure nitrocellulose membrane (Amersham). After blocking with nonfat milk buffer, the membrane was incubated with rabbit-anti-CD163 (1:2,000, Ab182422, Abcam), and mouse-anti-β-actin (1:50,000, A3854, Sigma). The protein bands were visualized and exposed to Kodak X-OMAT film. The OD value of each band was analyzed with Image J software and CD163 protein levels presented as CD163 to β-actin ratio.

### Statistical analysis

All measurements were conducted by investigators blinded to treatment assignment and the values of each group were presented as means ± SD. Statistically differences among groups were analyzed by one-way ANOVA. Statistically differences were deemed significant if *p* < 0.05.

## Results

**Figure 1:**
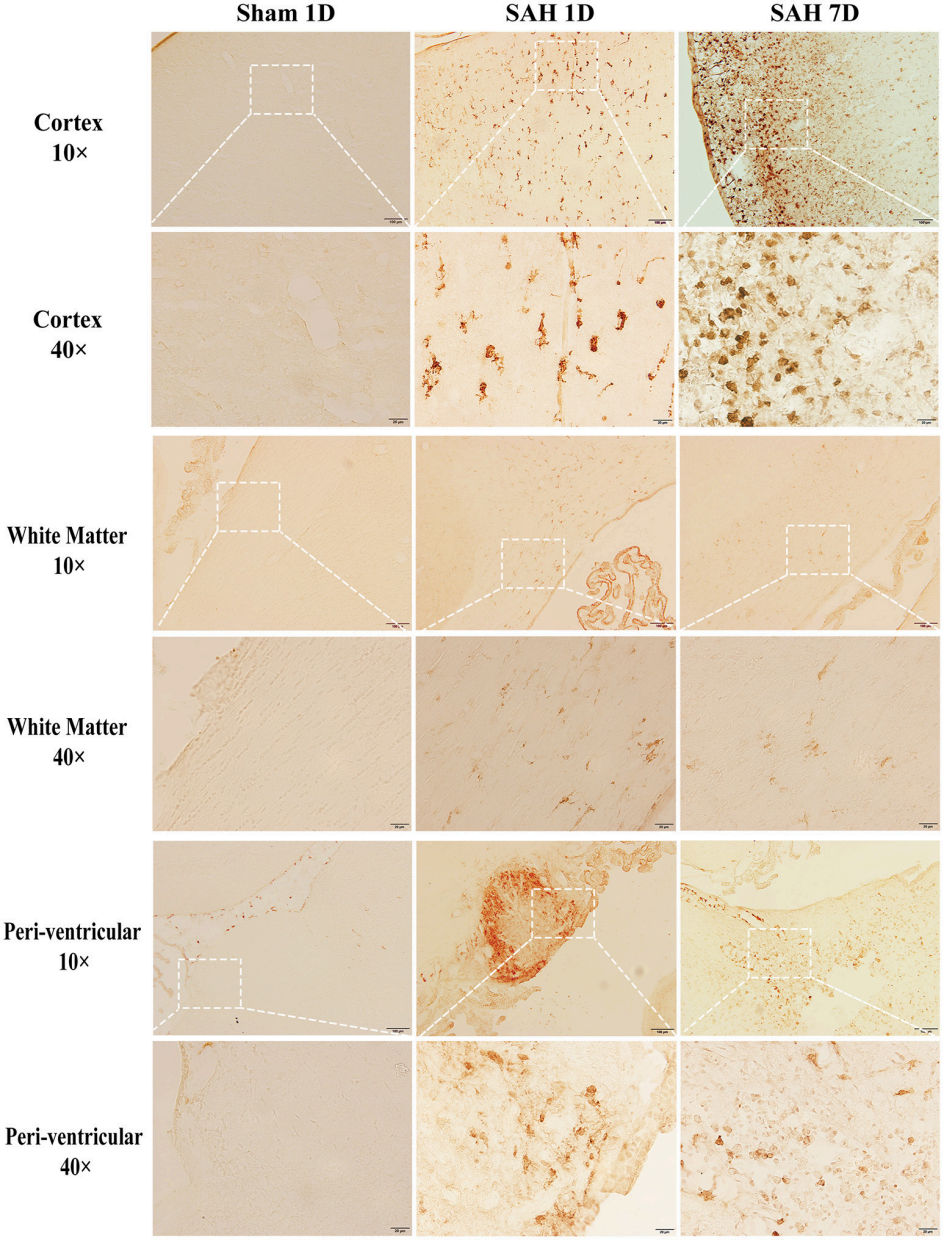
CD163 immunoreactivity in cortex, white matter (WM) and periventricular area at days 1 and 7 after SAH or a sham operation. Low power (x10, scale bar = 100 μm) and high power (x40, scale bar = 20 μm) images are shown. The latter correspond to the box areas in the lower power images.

**Figure 2:**
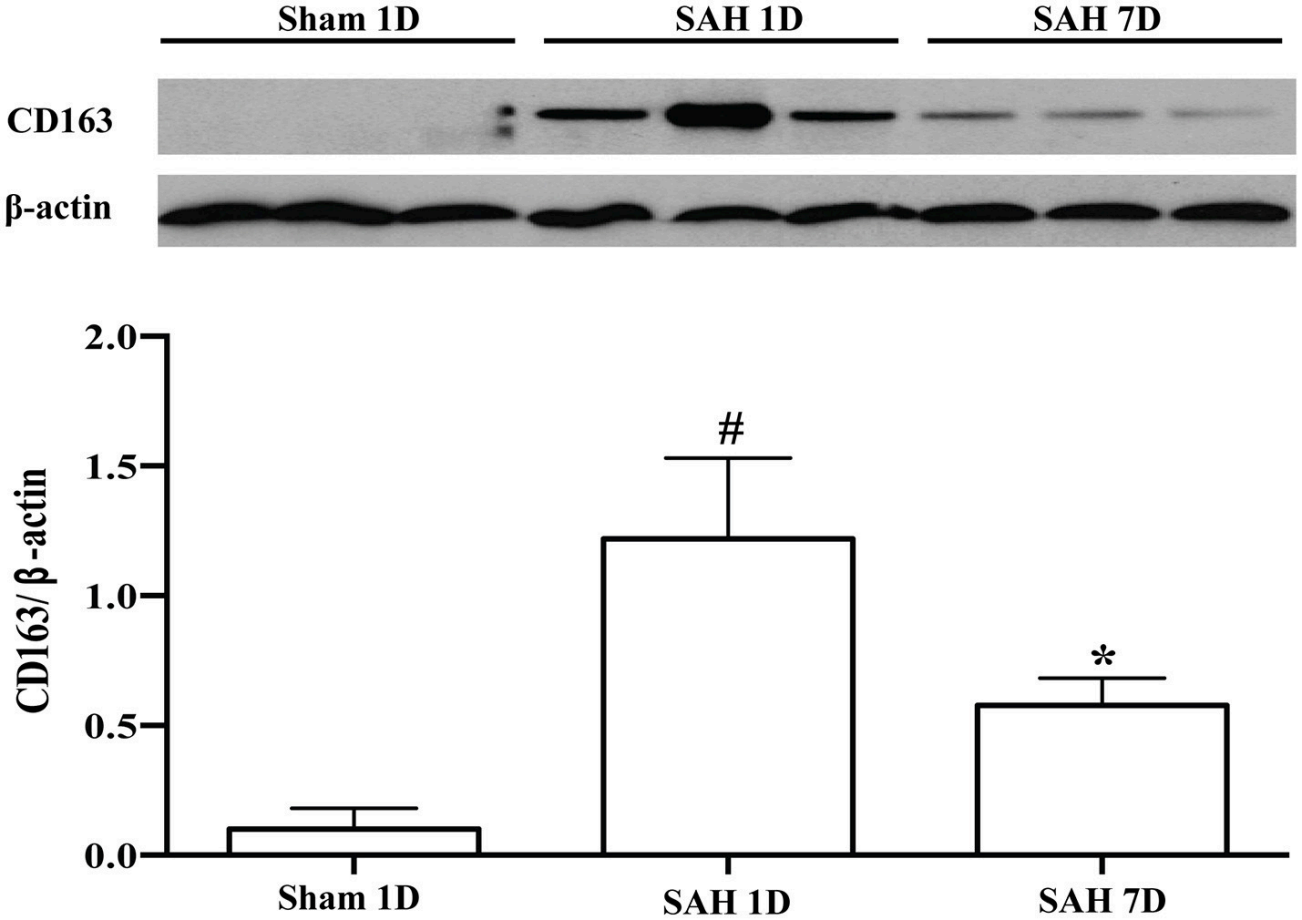
CD163 expression in ipsilateral cortex after SAH (days 1 and 7) or a sham operation (day 1) as assessed by Western blot. Values are means ± SD, ^*^p < 0.05 vs. sham; ^#^*p* < 0.01 vs. sham, *n* = 3, one-way ANOVA.

Brain injury and CD163 expression in the brain after SAH were diffuse. We used immunostaining to show the locations and cell types which express CD163. Brain CD163 levels were quantified by Western blots. One day after a sham operation, brain CD163 immunoreactivity was very low. In contrast, there were numerous CD163 positive cells in cortex, white matter and periventricular regions from days 1 to 7 after SAH (Figure 1). By Western blot, CD163 protein levels in ipsilateral cortex were upregulated after SAH, peaking at day 1(CD163/β-actin: 1.22 ± 0.31 vs. 0.09 ± 0.06 in sham, *p* < 0.01). However, high levels were still found 7 days after SAH (CD163/β-actin: 0.58 ± 0.10 vs. 0.09 ± 0.06 in sham, *p* < 0.05; Figure 2).

**Figure 3:**
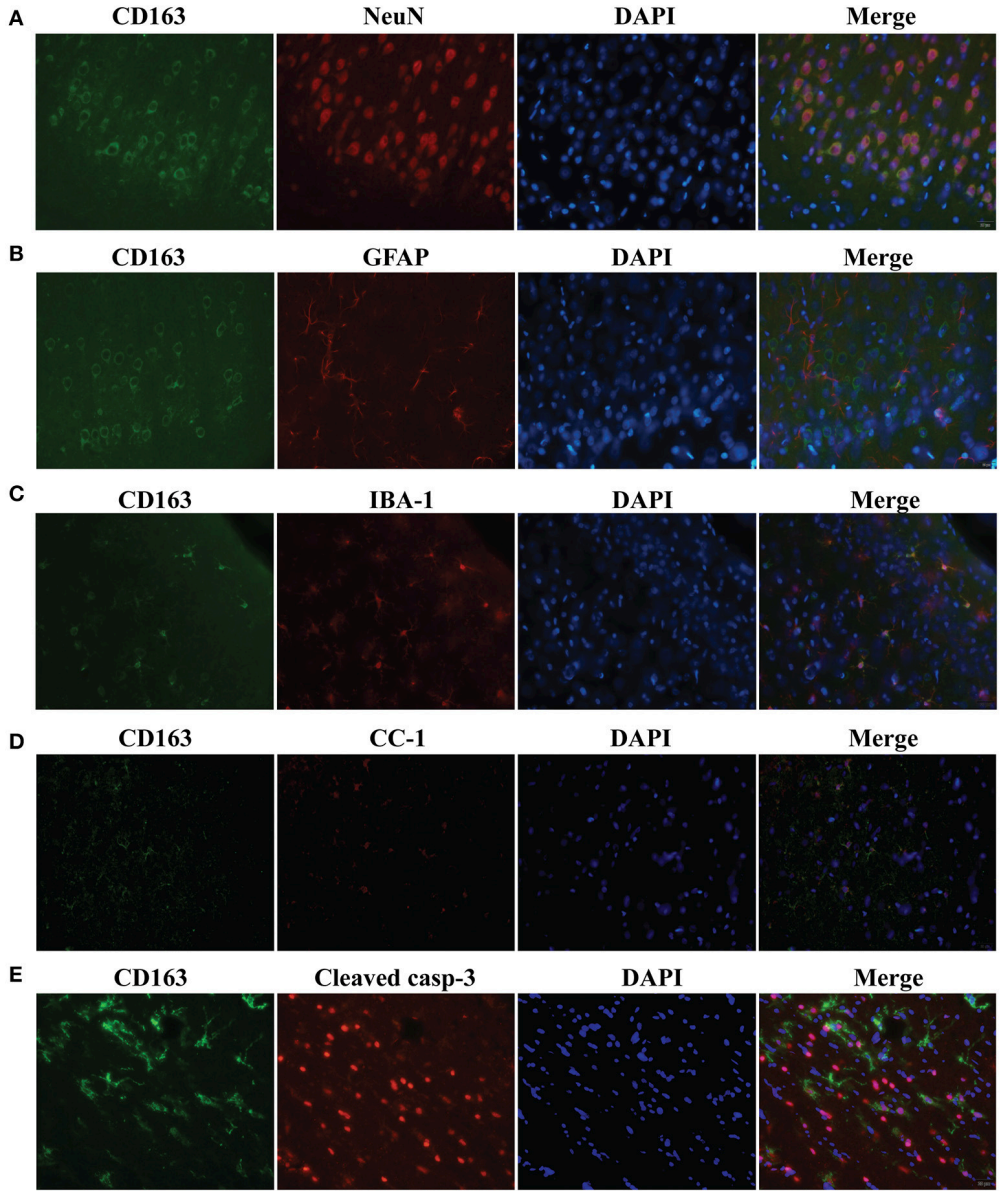
Triple immunofluorescence staining with CD163, NeuN **(A)**, IBA-1 **(B)**, GFAP **(C)**, CC1 **(D)** and cleaved caspase-3 **(E)** at day 1 following SAH. Scale bar = 20 μm.

To examine CD163 expression in specific cell populations, triple immunofluorescence labeling was carried out 24 h following SAH. Cells that stained positively for CD163 co-localized with NeuN-(a neuronal nuclei label, Figure 3A), IBA-1-(a microglia/macrophage marker, Figure 3C), CC1-(an oligodendrocyte marker, Figure 3D) and cleaved caspase-3-(a marker of apoptosis, Figure 3E) positive cells. However, it did but not co-localize with GFAP-(an astrocyte label, Figure 3B) positive cells. Thus, CD163 protein was expressed by neurons, microglia/macrophages and oligodendrocytes after SAH, but not astrocytes. Cleaved caspase-3-positive cells were found mainly in superficial cortex and white matter, indicating apoptosis is occurred in those cells, some of which expressed CD163 protein.

**Figure 4:**
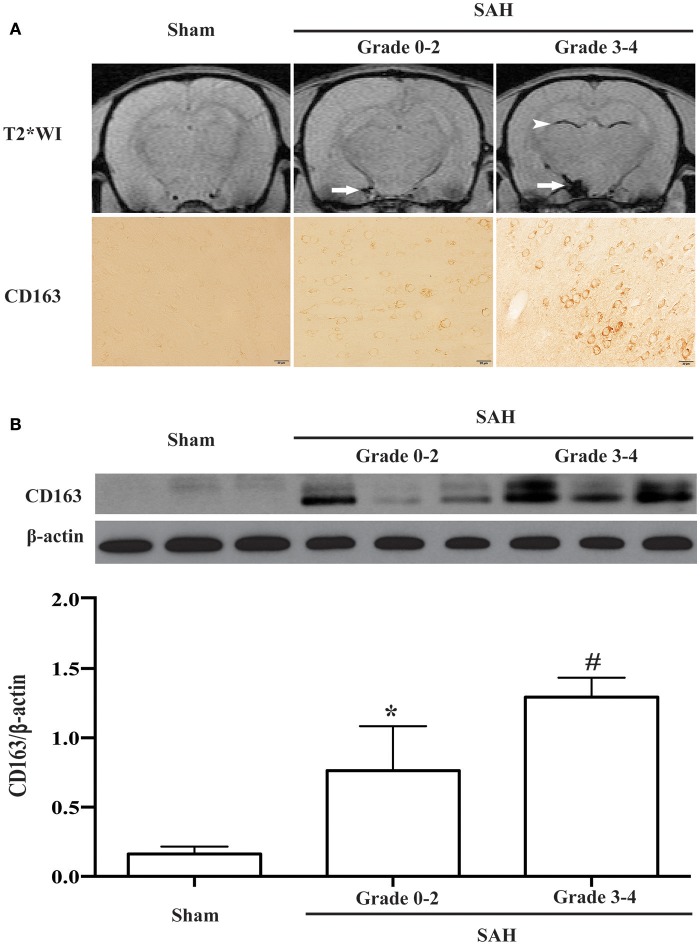
**(A)** Representative T2^*^ weighted MRI for SAH grading and corresponding CD163 immunoreactivity at day 1 following SAH or a sham operation. On the T2^*^ weighted images (T2WI), arrows point to the subarachnoid blood masses and arrowheads point to intraventricular blood clots. Scale bar = 20 μm. **(B)** CD163 expression in the ipsilateral cortex at day 1 following SAH of different grades (0-2 or 3-4) or a sham operation as assessed by Western blot. Values are means±SD, ^*^p < 0.05 grade 0-2 vs. the other groups; ^#^*p* < 0.01 grade 3-4 vs. sham, *n* = 3, one-way ANOVA.

All experimental rats were sorted on the basis of MRI grading. CD163-positive cells were found in SAH rats graded from 0 to 4 (Figure 4A). Compared with sham-operated and grades 0-2 (mild SAH) animals, the rats with grades 3-4 (severe SAH) had higher CD163 protein levels (CD163/β-actin: 1.29 ± 0.14 vs. 0.16 ± 0.05 in sham operated animals, *p* < 0.01; 0.76 ± 0.33 in grade 0-2 SAH, *p* < 0.05; Figure 4B).

**Figure 5:**
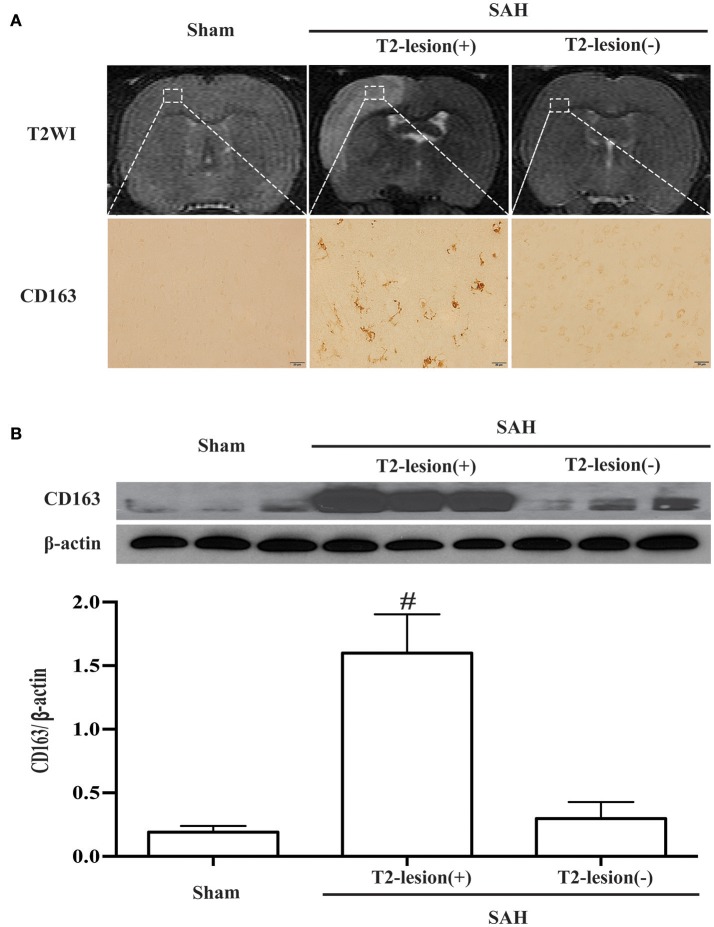
SAH-induced lesions were identified on T2 weighted MRI (T2WI). **(A)** Representative example of a T2 lesion 1 day after SAH [T2-lesion (+)] compared to images from a sham-operated rat and an SAH rat with no lesion [T2-lesion (–)]. Corresponding CD163 immunoreactivity in ipsilateral cortex from the box area is shown below. Scale bar = 20 μm. **(B)** Western blots were used to quantify CD163 protein levels in the ipsilateral cortex at day 1 in sham operated and SAH rats with and without a T2 lesion. Values are mean±SD, ^#^*p* < 0.01, T2-lesion (+) vs. the other groups, *n* = 3, one-way ANOVA.

T2 hyper-intensity areas in the ipsilateral cortex were found on T2 weighted images, and there were abundant CD163-positive cells in the T2 lesion (+) areas (Figure 5A). Accordingly, a marked increase of CD163 protein level was observed in T2 lesion (+) areas compared with sham-operated and T2 lesion (–) animals (CD163/β-actin: 1.60 ± 0.31 vs. 0.19 ± 0.05 in the control, *p* < 0.01; 0.30 ± 0.13 in T2 lesion (–) areas, *p* < 0.01; Figure 5B).

**Figure 6:**
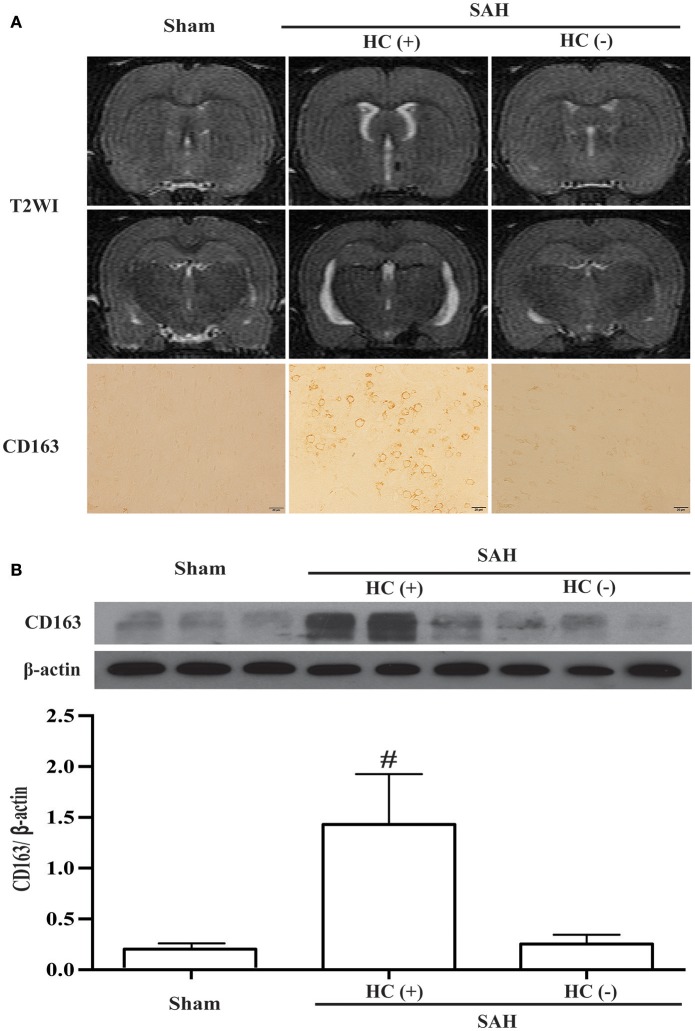
SAH-induced hydrocephalus was identified on T2 weighted MRI (T2WI). **(A)** Representative image of hydrocephalus [HC (+)] 1 day after SAH, note the dilated lateral ventricles. For comparison, images are shown of an animal with no hydrocephalus after SAH [HC (–)] and a sham-operated rat at day 1. Corresponding CD163 immunoreactivity in the ipsilateral cortex is shown below; scale bar = 20 μm. **(B)** Western blots were used to quantify CD163 protein levels in the ipsilateral cortex at day 1 in sham operated and SAH rats with and without hydrocephalus. Values are means±SD, ^#^*p* < 0.01, HC (+) vs. the other groups, *n* = 3, one-way ANOVA.

Hydrocephalus was also observed on T2 weighted images 24 h following SAH. Moreover, the SAH rats with hydrocephalus had higher CD163 immunoreactivity (Figure 6A). The CD163 expression assessed by Western blotting was higher in SAH rats with hydrocephalus compared with sham-operated and with no hydrocephalus animals (CD163/β-actin: 1.43 ± 0.49 vs. 0.18 ± 0.05 in the control, *p* < 0.01; 0.25 ± 0.09 in hydrocephalus (–) animals, *p* < 0.01; Figure 6B).

## Discussion

The main findings from our study are as listed below: (1) CD163 protein levels were elevated in rat brain following SAH; (2) CD163 protein was expressed by neurons, microglia/macrophages, and oligodendrocytes; (3) CD163-positive cells were also cleaved caspase-3-positive, indicating that CD163 positive cells could be apoptotic cells; and (4) CD163 overexpression was associated with severe hemorrhage, T2 lesion on MRI and hydrocephalus after SAH.

After SAH, hemoglobin is released through red cell lysis and it is important to understand how hemoglobin can be cleared. CD163 is a primary receptor for hemoglobin endocytosis and degradation. Our study found that CD163 expression rapidly increased in cortex, white matter and periventricular regions from days 1 to 7 following SAH. This response might be associated with penetration of hemoglobin into adjacent brain tissue from both the subarachnoid space and the ventricles (Turner et al., 1998). In microglia/macrophages, the CD163-mediated uptake is a high affinity pathway to eliminate extracellular hemoglobin and limit toxicity (Thomsen et al., 2013). However, our recent study also found that neurons could express CD163 after ICH (Liu et al., 2017) and there has been *in vitro* evidence that neuronal CD163 participates in hemoglobin-induced toxicity (Chen-Roetling and Regan, 2016). The difference in the effects of macrophage/microglia and neuronal CD163 may reflect the high ferritin expression in the former. The role of neuronal CD163 in SAH should be studied further.

Hemoglobin is degraded into heme which can then be metabolized by HO-1 into carbon monoxide, biliverdin, and free iron. Iron may then bind to ferritin, limiting iron-induced toxicity (Otterbein et al., 2003; Thomsen et al., 2013). Animal experimental studies have demonstrated that HO-1 protein expression is increased after SAH, followed by elevated free iron, transferrin, and ferritin levels, and that the iron chelator, deferoxamine, can reduce brain injury and neuronal death after SAH (Lee et al., 2010; LeBlanc et al., 2016; Guo et al., 2017). In view of above mentioned findings, it seems reasonable to speculate that the hemoglobin-CD163-HO-1 system is involved in acute brain damage following experimental SAH.

Cell apoptosis play an important role in early brain injury after SAH and is associated with poor outcomes (Lee et al., 2010; Sehba et al., 2012). Our data showed there was abundant co-localization of CD163 with cleaved caspase-3-positive cells. These findings suggest that CD163 may be associated with apoptotic cell death. Interestingly, CD163 expression is related with alternatively activated or anti-inflammatory microglia/macrophages that are found in the final resolution phase of inflammatory processes (Abraham and Drummond, 2006; Thomsen et al., 2013). Inflammation is considered to be important in the mechanisms of cell death and brain injury following SAH (Li et al., 2017; Zhang et al., 2017). Thus, the role of CD163 in hemoglobin uptake, inflammation, early brain injury and iron overload, as well as long-term neurologic deficits deserves further study.

It is well-known that neurologic deficits are related to the amount of blood released following SAH. In addition, we have found that SAH-induced acute hydrocephalus and white matter injury are also associated with SAH severity (Okubo et al., 2013; Egashira et al., 2014). A MRI grading system has been developed to evaluate SAH severity without requiring euthanasia. We have found that SAH rats with grades 3-4 had large T2 lesions, worse neurological outcomes, a higher incidence of hydrocephalus and white matter injury compared to grades 0-2 (Guo et al., 2017). The present study found that the T2 lesion (+) areas matched with CD163 positively labeled areas at day 1 after SAH. Our previous study has demonstrated that HO-1 protein levels were also significantly increased in T2 lesion areas (Guo et al., 2017), indicating that iron overload may be a leading cause of T2 lesion formation.

There were limitations in this study: (1) A causal relationship between CD163 expression and SAH-induced brain injury was not determined in the current study; (2) CD163 expression after SAH only determined at days 1 and 7. CD163 might be involved in early brain injury and delayed brain recovery. In future studies, a full time-course of CD163 expression in the brain following SAH should be examined.

## Conclusions

In summary, brain CD163 levels were increased in the acute phase of SAH, which was associated with hemorrhage severity, T2 lesion on MRI and hydrocephalus after SAH. The role of CD163 (beneficial or detrimental) in SAH remains to be determined.

## Author contributions

Immunostaining, Western blotting, data analysis, drafted the manuscript: CJ. Designed the experiment and data analysis: YH, RK. Animal surgery: HS, HZ.

### Conflict of interest statement

The authors declare that the research was conducted in the absence of any commercial or financial relationships that could be construed as a potential conflict of interest.

## References

[B1] Abraham N. G. Drummond G. (2006). CD163-Mediated hemoglobin-heme uptake activates macrophage HO-1, providing an antiinflammatory function. Circ. Res. 99, 911–914. 10.1161/01.RES.0000249616.10603.d6 17068296

[B2] Cao S. Zheng M. Hua Y. Chen G. Keep R. F. Xi G. (2016). Hematoma changes during clot resolution after experimental intracerebral hemorrhage. Stroke 47, 1626–1631. 10.1161/STROKEAHA.116.013146 27125525 PMC5237112

[B3] Chen-Roetling J. Regan R. F. (2016). Haptoglobin increases the vulnerability of CD163-expressing neurons to hemoglobin. J. Neurochem. 139, 586–595. 10.1111/jnc.13720 27364920 PMC5304903

[B4] Egashira Y. Hua Y. Keep R. F. Xi G. (2014). Acute white matter injury after experimental subarachnoid hemorrhage: potential role of lipocalin 2. Stroke 45, 2141–2143. 10.1161/STROKEAHA.114.005307 24893611 PMC4074774

[B5] Guo D. Wilkinson D. A. Thompson B. G. Pandey A. S. Keep R. F. Xi G.. (2017). MRI Characterization in the Acute Phase of Experimental Subarachnoid Hemorrhage. Transl. Stroke Res. 8, 234–243. 10.1007/s12975-016-0511-5 27896625 PMC5436945

[B6] Jing C. H. Wang L. Liu P. P. Wu C. Ruan D. Chen G. (2012). Autophagy activation is associated with neuroprotection against apoptosis via a mitochondrial pathway in a rat model of subarachnoid hemorrhage. Neuroscience 213, 144–153. 10.1016/j.neuroscience.2012.03.055 22521819

[B7] Kooijman E. Nijboer C. H. van Velthoven C. T. Kavelaars A. Kesecioglu J. Heijnen C. J. (2014). The rodent endovascular puncture model of subarachnoid hemorrhage: mechanisms of brain damage and therapeutic strategies. J. Neuroinflammation 11:2 10.1186/1742-2094-11-2 24386932 PMC3892045

[B8] LeBlanc R. H. Chen R. Selim M. H. Hanafy K. A. (2016). Heme oxygenase-1-mediated neuroprotection in subarachnoid hemorrhage via intracerebroventricular deferoxamine. J. Neuroinflammation 13:244 10.1186/s12974-016-0709-1 27618864 PMC5020472

[B9] Leclerc J. L. Lampert A. S. Loyola Amador C. Schlakman B. Vasilopoulos T. Svendsen P.. (2018). The absence of the CD163 receptor has distinct temporal influences on intracerebral hemorrhage outcomes. J. Cereb. Blood Flow Metab. 38, 262-273. 10.1177/0271678X17701459 28358264 PMC5951015

[B10] Lee J. Y. Keep R. F. He Y. Sagher O. Hua Y. Xi G. (2010). Hemoglobin and iron handling in brain after subarachnoid hemorrhage and the effect of deferoxamine on early brain injury. J. Cereb. Blood Flow Metab. 30, 1793–1803. 10.1038/jcbfm.2010.137 20736956 PMC2970675

[B11] Lee J. Y. Sagher O. Keep R. Hua Y. Xi G. (2009). Comparison of experimental rat models of early brain injury after subarachnoid hemorrhage. Neurosurgery 65, 331–343; discussion 343. 10.1227/01.NEU.0000345649.78556.26 19625913

[B12] Li J. R. Xu H. Z. Nie S. Peng Y. C. Fan L. F. Wang Z. J. (2017). Fluoxetine-enhanced autophagy ameliorates early brain injury via inhibition of NLRP3 inflammasome activation following subrachnoid hemorrhage in rats. J. Neuroinflammation 14:186 10.1186/s12974-017-0959-6 28903766 PMC5598033

[B13] Liu R. Cao S. Hua Y. Keep R. F. Huang Y. Xi G. (2017). CD163 Expression in neurons after experimental intracerebral hemorrhage. Stroke 48, 1369–1375. 10.1161/STROKEAHA.117.016850 28360115 PMC5404936

[B14] Madsen M. Graversen J. H. Moestrup S. K. (2001). Haptoglobin and CD163: captor and receptor gating hemoglobin to macrophage lysosomes. Redox Rep. 6, 386–388. 10.1179/135100001101536490 11865982

[B15] Okubo S. Strahle J. Keep R. F. Hua Y. Xi G. (2013). Subarachnoid hemorrhage-induced hydrocephalus in rats. Stroke 44, 547–550. 10.1161/STROKEAHA.112.662312 23212164 PMC3552015

[B16] Otterbein L. E. Soares M. P. Yamashita K. Bach F. H. (2003). Heme oxygenase-1: unleashing the protective properties of heme. Trends Immunol. 24, 449-455. 10.1016/S1471-4906(03)00181-9 12909459

[B17] Sehba F. A. (2014). Rat endovascular perforation model. Transl. Stroke Res. 5, 660–668. 10.1007/s12975-014-0368-4 25213427 PMC4214882

[B18] Sehba F. A. Bederson J. B. (2006). Mechanisms of acute brain injury after subarachnoid hemorrhage. Neurol. Res. 28, 381–398. 10.1179/016164106X114991 16759442

[B19] Sehba F. A. Hou J. Pluta R. M. Zhang J. H. (2012). The importance of early brain injury after subarachnoid hemorrhage. Prog. Neurobiol. 97, 14–37. 10.1016/j.pneurobio.2012.02.003 22414893 PMC3327829

[B20] Shishido H. Egashira Y. Okubo S. Zhang H. Hua Y. Keep R. F.. (2015). A magnetic resonance imaging grading system for subarachnoid hemorrhage severity in a rat model. J. Neurosci. Methods 243, 115–119. 10.1016/j.jneumeth.2015.01.035 25677406 PMC4359648

[B21] Thomsen J. H. Etzerodt A. Svendsen P. Moestrup S. K. (2013). The haptoglobin-CD163-heme oxygenase-1 pathway for hemoglobin scavenging. Oxid. Med. Cell. Longev. 2013:523652 10.1155/2013/523652 23781295 PMC3678498

[B22] Turner C. P. Bergeron M. Matz P. Zegna A. Noble L. J. Panter S. S.. (1998). Heme oxygenase-1 is induced in glia throughout brain by subarachnoid hemoglobin. J. Cereb. Blood Flow Metab. 18, 257–273. 10.1097/00004647-199803000-00004 9498842

[B23] Xi G. Keep R. F. Hoff J. T. (2006). Mechanisms of brain injury after intracerebral haemorrhage. Lancet Neurol. 5, 53–63. 10.1016/S1474-4422(05)70283-0 16361023

[B24] Zhang X. Wu Q. Zhang Q. Lu Y. Liu J. Li W. (2017). Resveratrol attenuates early brain injury after experimental subarachnoid hemorrhage via inhibition of NLRP3 inflammasome activation. Front. Neurosci. 11:611 10.3389/fnins.2017.00611 29163015 PMC5675880

